# Papillome inversé: à propos de 13 cas au Service d’Oto-Rhino-Laryngologie du Centre Hospitalier National Universitaire de Fann

**DOI:** 10.11604/pamj.2019.34.71.19888

**Published:** 2019-10-04

**Authors:** Moustapha Ndiaye, Ciré Ndiaye, Mame Sanou Diouf, Ndèye Oumy Sarr, Abdou Sy, Malick Ndiaye, Abdourahmane Tall

**Affiliations:** 1 Service d'Oto-Rhino-Laryngologie, Centre Hospitalier National Universitaire de Fann, Dakar, Sénégal; 2 Service d'Oto-Rhino-Laryngologie, Hôpital d'Enfants de Diamniadio, Dakar, Sénégal

**Keywords:** Papillome inversé, traitement endoscopique endonasal, CHNU FANN, Inverted papilloma, endoscopic endonasal treatment, National University Hospital of Fann

## Abstract

**Introduction:**

Le Papillome Inversé (PI) est une tumeur bénigne nasosinusienne rare caractérisée histologiquement par une invagination de l'épithélium de surface dans le chorion sous-jacent. Elle se distingue par une forte agressivité locale, une tendance à la récidive et par son risque imprévisible d'association à un carcinome épidermoïde. L'objectif de l'étude était de rapporter les données épidémiologiques, cliniques, paracliniques ainsi que d'évaluer les résultats de la chirurgie endoscopique endonasale.

**Méthodes:**

Il s'agit d'une étude rétrospective portant sur une série de 13 patients colligés au Service d'Oto-Rhino-Laryngologie du centre hospitalier national universitaire de Fann, du 1^er^ janvier 2012 au 31 décembre 2017. Ont été inclus dans l'étude tous les patients suivis pour un papillome inversé confirmé à l'examen anatomopathologique.

**Résultats:**

L'âge moyen de nos patients était de 44 ans et le sex-ratio (H/F) de 2,25. Tous les patients avaient présenté une obstruction nasale alors que la rhinorrhée était présente dans 53% des cas suivis de l'épistaxis dans 30% des cas. La symptomatologie était latéralisée à droite dans 69% des cas, 23% à gauche et 7% de façon bilatérale. La rhinoscopie antérieure avait permis de visualiser une masse endonasale chez tous les patients. Tous les patients avaient bénéficié d'une TDM; l'IRM avait été réalisée chez un seul patient. L'exérèse du PI par voie endoscopique endonasale avait été effectuée chez 10 patients (76,9%) alors que la voie externe avait été utilisée dans 23% des cas. La chirurgie avait permis de préciser la base d'implantation de la tumeur qui était de l'ordre de 46% dans le sinus maxillaire, 15% dans le cornet inférieur, 15% dans le cornet moyen, 7% dans la bulle ethmoïdale et 7% dans la paroi latérale de la fosse nasale. Un patient avait eu une récidive du papillome inversé après un délai moyen de 26 mois. L'association maligne s'était révélée par un cas de dégénérescence maligne en carcinome épidermoïde.

**Conclusion:**

Le papillome inversé est une tumeur très agressive. La TDM fournit beaucoup de renseignements à propos de son extension, surtout osseuse. Le traitement est actuellement révolutionné par la chirurgie endoscopique qui offre d'excellents résultats. Mais, il existe néanmoins un risque de récidive après chirurgie qui doit motiver une surveillance au long cours.

## Abstract (en)

**Introduction:**

Inverted Papilloma (IP) is a rare benign tumour of the nose and paranasal sinuses histologically characterized by invagination of the outer layer of the epithelium in the underlying chorion. Its most distinctive feature is a strong local aggressiveness, a tendency to recur and an unpredictable risk of association with epidermoid carcinoma. The purpose of this study was to report the epidemiological, clinical, paraclinical data as well as to assess the outcomes of endoscopic endonasal surgery.

**Methods:**

We conducted a retrospective study on a series of 13 patients whose data were collected in the Oto-Rhino-Laryngology at the National University Hospital Center of Fann, from 1^st^ January 2012 to 31^st^ December 2017. All patients followed up for inverted papilloma confirmed by anathomopathological examination were included in the study.

**Results:**

The average age of patients was 44 years, the sex-ratio was 2.25. All patients had nasal obstruction; 53% of them had rhinorrhea followed by epistaxis in 30% of cases. Right-sided symptoms were reported by 69% of patients, left-sided symptoms by 23% and bilateral symptoms by 7% of patients. Anterior rhinoscopy showed endonasal mass in all patients. All patients underwent CT scan; the MRI was performed in a single patient. Endoscopic resection of IP was performed in 10 patients (76.9%) while external approach was used in 23% of cases. Surgery helped to clarify where the tumor had originated: in 46% of cases in the maxillary sinus, in 15% in the lower nasal turbinate, in 15% in the mid-turbinate, in 7% in the ethmoid bulla and in 7% in the lateral wall of the nasal fossa. One patient had recurrence after an average period of 26 months. Malignant transformation to squamous cell carcinoma occurred in one patient.

**Conclusion:**

Inverted papilloma is a very aggressive tumor. CT scan is essential to highlight its spread, especially to bone. The advent of endoscopic surgery has revolutionised treatment, providing excellent results. But, there is nevertheless a risk of recurrence after surgery which motivates an indication for long term monitoring.

## Introduction

Le papillome inversé nasosinusien est une tumeur bénigne qui présente des similitudes avec les tumeurs malignes: forte agressivité locale et tendance à la récidive. La notion de dégénérescence est aussi vérifiée. La TDM et l'IRM apprécient l'extension mais le diagnostic est histologique. A travers une revue de la littérature, nous allons partager notre expérience sur les 13 cas colligés en 06 ans.

## Méthodes

Il s'agit d'une étude rétrospective portant sur une série de 13 patients colligés au Service d'Oto-Rhino-Laryngologie du Centre Hospitalier National Universitaire de Fann, du 1^er^ janvier 2012 au 31 décembre 2017. Ont été inclus dans l'étude tous les patients suivis pour un papillome inversé confirmé à l'examen anatomopathologique. Tous ces patients ont été pris en charge chirurgicalement.

## Résultats

**Figure 1:**
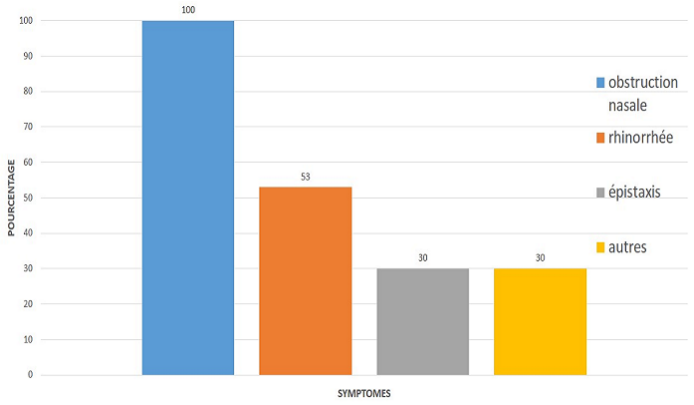
Répartition des symptômes

**Figure 2:**
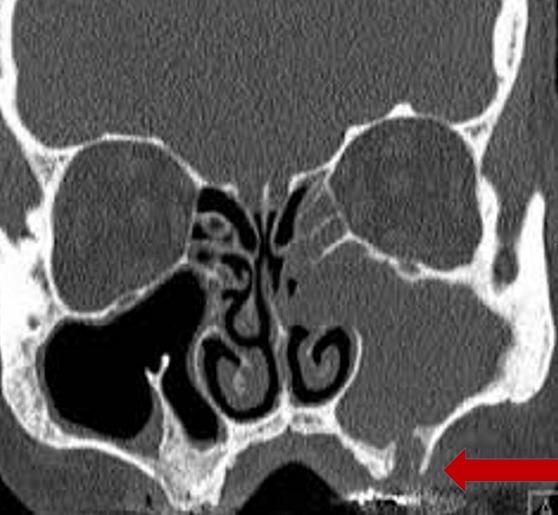
TDM en coupe coronale d’un PI ethmoïdo-maxillaire gauche: lyse de la paroi inférieure du sinus maxillaire (flèche rouge)

L'incidence était de 2 patients/an. L'âge moyen de nos patients était de 44 ans avec des extrêmes allant de 28 à 74 ans. Le sex-ratio (H/F) était de 2,25. Le délai moyen de consultation était de 09 ans. Dans les antécédents médico-chirurgicaux, on notait 1 cas de papillome inversé opéré en 2009 en France; le geste consistait à une maxillectomie médiale. Trois patients étaient tabagiques, la notion d'allergie nasosinusienne était retrouvée chez un patient. Tous les patients avaient présenté une obstruction nasale alors que la rhinorrhée était présente dans 53% des cas suivis de l'épistaxis dans 30% des cas. La symptomatologie était latéralisée à droite dans 69% des cas, 23% à gauche et 7% de façon bilatérale. Les autres motifs de consultation étaient à type de céphalées, algies cranio-faciales et hyposmie. La Figure 1 résume l'ensemble des symptômes recueillis. La rhinoscopie antérieure avait permis de visualiser une masse endonasale chez tous les patients. Cette masse était d'aspect cérébriforme dans 46% des cas, bourgeonnante rosée dans 23%, papillomateuse dans 15% et polypoïde dans 15%. Onze patients avaient bénéficié d'une TDM; l'IRM avait été réalisée chez un seul patient. La TDM (Figure 2) montrait une formation de densité tissulaire qui comblait les sinus. On notait 4 cas de lyse osseuse (3,33%) qui intéressaient les parois des sinus maxillaire, sphénoïdal et ethmoïdal ainsi que la cloison nasale. Les sites d'extension sont résumés dans le Tableau 1.

**Tableau 1:** Récapitulatif des différents sites d’extension du papillome inversé

Site	N
Sinus Maxillaire	11
Sinus Frontal	03
Sinus Ethmoidal	07
Fosses Nasales	13
Ethmoide	04

L'exérèse du PI par voie endoscopique endonasale avait été effectuée chez 10 patients (76,9%) alors que la voie externe avait été utilisée dans 23% des cas. La chirurgie avait permis de préciser la base d'implantation de la tumeur qui était de 46% dans le sinus maxillaire, 15% dans le cornet inférieur, 15% dans le cornet moyen, 7% dans la bulle ethmoïdale et 7% dans la paroi latérale de la fosse nasale. Concernant la chirurgie endoscopique, les gestes réalisés en fonction du site d'implantation et de l'extension de la tumeur avaient été: 06 cas d'éthmoïdectomie antérieure, 05 cas de méatotomie moyenne et 03 cas de turbinectomie inférieure. Le méchage des fosses nasales avait été effectué chez tous les patients et gardé pendant 2 jours. Les complications étaient à type d'œdème palpébral (1 cas) et de synéchie entre le cornet inférieur et la cloison nasale (1 cas). Un patient opéré par voie endonasale avait eu une récidive du papillome inversé après un délai moyen de 26 mois; l'atteinte concernait le sinus maxillaire. L'association maligne s'etait révélée par un cas de dégénérescence maligne en carcinome épidermoïde. Les examens anatomopathologiques effectués sur les pièces opératoires avaient montré un aspect de papillome inversé dans tous les cas: formations papillaires faites d'un axe conjonctif tapissé d'un épithélium malpighien kératinisé avec la présence de prolongements endophytiques dans le chorion sous-jacent. La membrane basale était intacte chez tous les patients à l'exception de celui qui avait une dégénérescence maligne.

## Discussion

Fort heureusement la tumeur agressive qu'est le PI est une pathologie rare. La fréquence varie entre 0,5 à 4% des tumeurs primitives des cavités nasosinusienne [1, 2]. L'incidence du PI varie de 0,2 à 1,5/100.000 habitants/an [2, 3]. Cette rareté de la pathologie est vérifiée dans notre étude (13 cas en 06 ans). L'âge moyen de 44 ans retrouvé dans notre étude corrobore les résultats de la littérature. Le PI se rencontre chez l'adulte âgé de 50 ans environ [4-6]. Plusieurs études rapportent une prédominance masculine [7, 8] comme c'est le cas pour notre étude; cette constatation reste inexpliquée. Dans la littérature, le délai de consultation varie de 12 à 66 mois [8-10]. Dans notre étude, ce délai est largement dépassé (9 ans); la lenteur d'évolution du PI ainsi que la difficulté d'accès aux soins dans nos contrées pourraient expliquer ce fait. Les facteurs étiologiques demeurent inconnus. Si certains auteurs émettent l'hypothèse du tabagisme, de l'allergie et de l'exposition virale [3, 11, 12] d'autres incriminent le Human Papilloma Virus (HPV) [8, 13]. Le caractère récidivant du PI ainsi que son potentiel d'évolution carcinomateuse jouerait en faveur de cette origine virale.

La symptomatologie est dominée par l'obstruction nasale suivie de la rhinorrhée alors que l'épistaxis dépasse rarement les 50% [4]. L'examen des fosses nasales retrouve une tumeur de couleur grise-rougeâtre, de consistance plus ferme que les polypes inflammatoires, d'aspect lobulé, présentant ainsi un aspect framboisé assez caractéristique. À la palpation, le PI est classiquement friable et saignant au contact [14]. Barnes, décrivait un aspect cérébriforme très caractéristique du PI. L'étude histologique effectuée après une biopsie minutieuse en profondeur permet de poser le diagnostic de PI, de rechercher des stigmates d'infection à HPV ou des foyers carcinomateux. Les prélèvements biopsiques insuffisants exposent à des risques de faux négatifs qui sont évalués à 17% [14]. La présence de stigmates d'infection à HPV varie entre 10 et 75% [15] alors que l'association carcinomateuse se situe entre 1 à 53% [16]. La TDM est l'examen clé du diagnostic. Elle permet d'apprécier l'extension et de prédire le site d'implantation du PI. Les 2 principaux sites d'extension sont les sinus maxillaire et ethmoïdal [17] comme dans notre étude. La valeur prédictive positive (VPP) du site d'insertion du PI en fonction de l'hyperostose est estimée entre 89% et 95% selon les études [14]. Dans notre étude, l'appréciation du site d'implantation à la TDM a été difficile du fait de l'aspect volumineux des tumeurs rencontrées. La présence de micro calcifications (20% des cas) [14] serait liée à un phénomène de piégeage de séquestres osseux, dû au remaniement inflammatoire [18]. L'IRM vient en complément à la TDM car permet de distinguer tumeur et inflammation. Dans notre contexte de pays en développement, l'IRM est très peu accessible justifiant sa faible réalisation.

La prise en charge est chirurgicale. La voie d'abord peut être externe ou endonasale sous guidage endoscopique selon la localisation de la tumeur et l'expérience du chirurgien. La voie externe ou voie de rhinotomie paranasale permet de bien exposer la tumeur et d'avoir un bon contrôle sur l'exérèse. Elle est préconisée en cas de PI étendue et permet de limiter les récidives [19, 20]. La voie d'abord endonasale sous guidage endoscopique est la nouvelle technique adoptée depuis les années 1990 et représente le gold standard [16]. Cette voie d'abord connait cependant des limites en cas d'extension aux sinus frontaux et maxillaires; le taux de récidive y serait de 70% [21]. D'ailleurs, notre seul cas de récidive concernait une atteinte du sinus maxillaire. Certains auteurs préconisent la réalisation d'une maxillectomie médiale pour pouvoir contrôler n'importe quelle lésion du sinus maxillaire [22] alors que d'autres utilisent la voie de Caldwell-Luc en cas d'atteinte latéral du sinus maxillaire [23, 24]. Dans notre étude, le seul cas de récidive concernait la chirurgie par voie endoscopique. Le taux de récidive concernant la chirurgie endoscopique est évalué à 12% selon Busquets [25]. Les récidives précoces survenant avant 2 ans seraient liées à une exérèse incomplète alors que les récidives tardives seraient en rapport avec une étiologie virale [14]. Le taux de transformation maligne est évalué à 8% en moyenne [26].

## Conclusion

Le PI est une tumeur bénigne agressive dont l'étiologie reste inconnue. La chirurgie endoscopique endonasale représente le gold standard dans le cadre de sa prise en charge mais connait ses limites. La possibilité de récidive et de dégénérescence maligne doit inciter à une surveillance au long cours.

### Etat des connaissances actuelles sur le sujet

- Le papillome inversé nasosinusien est une tumeur bénigne agressive pouvant simuler parfois une tumeur maligne;
- Il a tendance à récidiver après exérèse et peut dégénérer en cancer;
- La chirurgie par voie endoscopique endonasale constitue le gold standard du traitement mais peut s'avérer insuffisante dans certaines situations obligeant ainsi un abord par voie externe complémentaire.

### Contribution de notre étude à la connaissance

- L'étude montre que l'examen clinique associé à la TDM permet d'évoquer le diagnostic de papillome inversé et de bien situer les extensions de la maladie même en l'absence de l'IRM. Cette attitude profite aux praticiens des pays en voie de développement où l'IRM fait défaut en général;
- La chirurgie par voie externe est désormais destinée aux tumeurs très étendues, difficile d'accès par voie endoscopique ou en cas de dégénérescence maligne.

## Conflits d’intérêts

Les auteurs ne déclarent aucun conflit d'intérêts.
